# Short chain acyl-CoA dehydrogenase deficiency and short-term high-fat diet perturb mitochondrial energy metabolism and transcriptional control of lipid-handling in liver

**DOI:** 10.1186/s12986-016-0075-0

**Published:** 2016-03-01

**Authors:** Sujoy Ghosh, Claudia Kruger, Shawna Wicks, Jacob Simon, K. Ganesh Kumar, William D. Johnson, Randall L. Mynatt, Robert C. Noland, Brenda K. Richards

**Affiliations:** Pennington Biomedical Research Center, Louisiana State University System, 6400 Perkins Road, Baton Rouge, LA 70808-4124 USA; Duke-NUS Graduate Medical School, Singapore, 169857 Singapore; Present Address: Taconic Biosciences, Inc., Germantown, NY USA

**Keywords:** Short chain acyl-CoA dehydrogenase deficiency, Fatty acid beta-oxidation, Mitochondrial

## Abstract

**Background:**

The liver is an important site of fat oxidation, which participates in the metabolic regulation of food intake. We showed previously that mice with genetically inactivated *Acads*, encoding short-chain acyl-CoA dehydrogenase (SCAD), shift food consumption away from fat and toward carbohydrate when tested in a macronutrient choice paradigm. This phenotypic eating behavior suggests a link between fat oxidation and nutrient choice which may involve an energy sensing mechanism. To identify hepatic processes that could trigger energy-related signals, we have now performed transcriptional, metabolite and physiological analyses in *Acads-/-* mice following short-term (2 days) exposure to either high- or low-fat diet.

**Methods and Results:**

Metabolite analysis revealed 25 acylcarnitine species that were altered by diet and/or genotype. Compared to wild-type mice, phosphorylated AMP-activated protein kinase was 40 % higher in *Acads-/-* mice after short-term high-fat diet, indicating a low ATP/AMP ratio. Metabolite analyses in isolated liver mitochondria from *Acads-/-* mice during ADP-linked respiration on butyrate demonstrated a reduced oxygen consumption rate (OCR) compared to wild-type, an effect that was not observed with succinate or palmitoylcarnitine substrates. Liver transcriptomic responses in *Acads-/-* mice fed high- vs. lowfat diet revealed increased RXR/PPARA signaling, up-regulation of lipid handling pathways (including beta and omega oxidation), and increased mRNA expression of Nfe2l2 target genes.

**Conclusions:**

Together, these results point to an oxidative shortage in this genetic model and support the hypothesis of a lower hepatic energy state associated with SCAD deficiency and high-fat diet.

**Electronic supplementary material:**

The online version of this article (doi:10.1186/s12986-016-0075-0) contains supplementary material, which is available to authorized users.

## Background

*Acads* encodes short-chain acyl-CoA dehydrogenase (SCAD), a member of the family of four enzymes that catalyzes the first of four sequential steps in the mitochondrial fatty acid oxidation spiral which produces acetyl-CoA for the tricarboxylic acid cycle. The optimum substrate for SCAD is butyryl-CoA, a fatty acid with four carbon units. Fatty acids are an important source of energy, particularly for tissues with high metabolic demands such as the liver, which plays a major role in the regulation of energy homeostasis in mammals.

Previously we reported that mice with a global, genetic inactivation of the gene encoding short-chain acyl-CoA dehydrogenase (*Acads−/−*) shift consumption away from fat and toward carbohydrate when offered a choice between macronutrient-rich diets [1]. As the mice avoid eating fat, they apparently compensate by eating more carbohydrate, thus preventing a reduction in caloric intake. Our observation provides evidence for a link between fatty acid oxidation and fat selection. Furthermore, the potential for altered fatty acid oxidation to affect the selection of fat-rich diets could be dependent on hepatic energy status, as changes in ATP/ADP ratio are quite likely sensed by the brain [2–5] to ultimately influence feeding behavior. Thus it is reasonable to postulate the liver as a site where signals related to fatty acid-derived energy may originate. Based on this proposition, the current study addressed the metabolic consequences of short-chain acyl-coenzyme A (CoA) dehydrogenase (SCAD) deficiency and dietary fat content on liver metabolism, including effects on tissue acylcarnitines, mitochondrial oxidative metabolism, hepatic pAMPK level, and genome-wide transcription. These studies were performed in the liver of *Acads*−/− and *Acads+/+* mice fed high- or low-fat diet for 2 days.

## Methods

### Animals and diets

The BALB/cByJ (*Acads−/−*) and BALB/cByKZ (*Acads+/+*) mice were obtained from breeding colonies maintained at the Pennington Biomedical Research Center (PBRC). Mice were singly housed in filter-top cages and kept in a specific-pathogen free facility, under 12 h light/12 h dark cycle and at an ambient temperature of 22–23 °C. All animals were fed standard rodent chow (no. 5001, LabDiets, Richmond, IN) until initiation of experimental diets. All animal experiments were approved by the Institutional Animal Care and Use Committee of the PBRC. The BALB/cByJ mouse strain carries a spontaneous 278-bp deletion at the 3’ end of the structural gene for *Acads* [6, 7], resulting in missplicing of RNA and a truncated, unstable SCAD enzyme with no residual activity [8]. This mutation occurred in the BALB/cByJ subline around 1982 [9] after it was separated from the BALB/cBy subline. Although the BALB/cByJ or *Acads−/−* mice have no detectable butyryl-CoA dehydrogenase activity [8], they appear to be normal [8, 10]. Their primary clinical phenotype is an impaired adaptation to prolonged fasting, due to the rapid depletion of hepatic glycogen stores [8]. The BALB/cBy line is considered the best *Acads+/+* control for the *Acads−/−* BALB/cByJ [11]. Our BALB/cByKZ.*Acads+/+* substrain was separated from the research colonies at The Jackson Laboratory in 1996.

Several days prior to each experiment, bedding was removed and replaced with stainless steel wire floor inserts, to permit measurement of spilled food. Polyvinylchloride nesting tubes were provided to reduce time spent on wire flooring. The experimental diets were equivalent for both micronutrients and for protein (16.4 % of energy). The balance of calories was contributed by 58 % fat and 25.5 % carbohydrate in D12331 and by 10.5 % fat and 73.1 % carbohydrate in D12329 (Research Diets, Inc., New Brunswick, NJ). The diets obtain most of their fat content from medium chain fatty acids. Tissues were harvested on day 2 after initiation of the high-fat (D12331) or low-fat (D12329) diet. Thus, there was no diet choice in the current study design. We selected the day 2 time point for tissue harvest to coincide with the time at which animals begin to avoid eating fat in a diet selection paradigm [1].

### Measurement of acylcarnitines

Twelve week old male BALB/cByJ (*Acads−/−*) and BALB/cByKZ (*Acads+/+*) mice were fed the experimental high- (HF) and low-fat (LF) diets for two days and then killed by CO_2_ inhalation followed by cervical dislocation. The liver was rapidly excised, frozen in liquid nitrogen, then powdered under liquid nitrogen and stored at −80 °C. Measurement of liver acylcarnitine content was performed by the Metabolomics Core of the University of Michigan. Liver samples were extracted and proteins precipitated by addition of solvent containing labeled internal standards (Cambridge Isotopes, labeled carnitine standards set B, cat#NSK-B1). Weighed samples were sonicated briefly (10 % duty cycle, 2 min, on ice) in the extraction solvent (Methanol-Chloroform-Water (8:1:1). Acylcarnitine species were measured by MRM transitions using an Agilent 1260 HPLC and 6430 Triple Quadrupole LC/MS system (Agilent Technologies, Santa Clara, CA). All data were acquired and analyzed using Agilent Masshunter Quantitative software, version 6.0. Standard curves were generated and acylcarnitines were measured using isotope dilution internal standards. Data were normalized to wet tissue weight and analyzed using 2-way analysis of variance in SAS v9.4 (SAS Institute Inc., Cary, NC, USA).

### Protein analysis by western blotting

Male, *Acads−/−* and *Acads+/+* mice were fed the HF and LF experimental diets for 0, 1, or 2 days. On the day of tissue harvest, mice were euthanized with CO_2_ between 10:00 and 11:00 am; the liver was quickly removed, frozen in liquid nitrogen and stored at −80 °C. Total protein was isolated using Lysis buffer (25 mM HEPES pH 7.8, 1 % Nonidet P-40, 50 mM KCl, 125 μM DTT, 1 mM PMSF, 5 mM NaPPi, 1 mM EDTA, 50 mM NaF including protease inhibitor cocktail). The samples were centrifuged and protein concentrations of the supernatants were determined using the BCA Protein Assay (Pierce Biotechnology, Rockford, IL). Electrophoretic separations were carried out on Mini-Protean II electrophoresis cells (Bio-Rad Laboratories, Inc., Hercules, CA). Supernatants (45 μg) were resolved by 10 % SDS-PAGE and subjected to immunoblotting. The protein was detected with antibodies against AMPKp(Thr^172^), AMPK (Abcam Inc., Cambridge, MA) and beta Actin (HRP) (Abcam) using Chemiluminescence Reagent Plus (Perkin-Elmer Life Sciences). Signal intensity was quantified by densitometry analysis using ImageJ Software [12].

### Preparation of mouse liver mitochondria

Male *Acads−/−* and *Acads+/+* mice were fed the high fat (HF) diet for two days, as described above. On the morning of the experiment, mice were euthanized using CO_2_ inhalation and liver tissue was collected in ice cold isolation buffer and subsequently homogenized according to the detailed method of Frezza et al. [13]. Mitochondrial protein content was measured using the BCA Protein Assay (Pierce Biotechnology, Rockford, IL). The preparation was kept on ice at all times and used within 6 h of isolation for mitochondrial bioenergetic measurements.

### Measurement of mitochondrial respiration

The oxygen consumption rate (OCR) of mitochondria during respiration was measured in the assay solution (MAS-1, 1X): 70 mM sucrose, 220 mM mannitol, 10 mM KH_2_PO_4_, 5 mM MgCl_2_, 2 mM HEPES, 1 mM EGTA and 0.2 % BSA, using a Seahorse XF24 Extracellular Flux Analyzer (Seahorse Bioscience, North Billerica MA, USA) [14]. Ten (10) μg of freshly prepared mitochondria were added to each well in an initial volume of 50ul, and the plate was centrifuged at 1800 g, 4 °C for 10 min to insure that mitochondria adhered to the bottom of the well. Substrate mix was added and the plate was brought to equilibrium at 37 °C for 10 min before loading it into the XF24-3 and initiating the experiment. State 4 respiration was determined in the presence of either succinate/rotenone (5.5 mM/2.2uM), butyrate/malate (900uM/2 mM), or palmitoylcarnitine/malate (80uM/2 mM) as substrates. The substrates butyrate/malate and palmitoylcarnitine/malate were used to involve short- and long-chain acyl-CoA dehydrogenases, respectively. First, two baseline measurements of oxygen consumption rate (OCR) were obtained. Next, ADP (4 mM) was injected to determine state 3 respiration, followed by sequential injections of oligomycin (2uM), FCCP (4uM) and antimycin A (4uM). Collectively, this injection series allowed for determination of ATP-linked OCR (ADP), proton leak (oligomycin), maximal respiration (FCCP) and non-mitochondrial or residual oxygen consumption (antimycin A). Samples from individual animals were run in 2–3 replicates using typical mix and measurement cycle times [14]. Respiratory control ratios (RCR) were calculated as the state 3 respiratory rate divided by the state 4 respiratory rate, obtained from the ADP-linked/oligomycin OCR readings [15]. Data are displayed as absolute, point-to-point oxygen consumption rates (pmol/min/well) and were analyzed using the XF software and Microsoft Excel. OCR data represent the mean of 2–3 replicate wells ± SE. Differences in OCR between genotype groups were tested using a two-tailed Student’s *t*-test, with *P* values less than 0.05 considered to be statistically significant.

### Measurement of mitochondrial DNA (mtDNA) content

Mitochondrial DNA was quantified by determining the ratios of encoded mitochondrial cytochrome c oxidase (*mt-Co2*) and cytochrome b (*mt-Cybt*) to nuclear intron of hemoglobin beta (*Hbb*) and glucagon (*Gcg*), respectively, by qRT–PCR of isolated DNA. Total DNA was extracted from liver using the DNeasy Qiagen Kit (Qiagen, Germantown, MD) and amplified using published primer sequences [16] and quantitative PCR (7900HT Sequence Detection System; Life Technologies, Carlsbad, CA). All primers were synthesized by Sigma-Aldrich (St. Louis, MO, USA).

### Microarray studies

Mice used for tissue harvest in the microarrays showed no statistically significant differences in baseline body weight or total energy intake between the genotype (*Acads−/−, Acads+/+*) or diet (HF, LF) groups (Additional file 1: Table S1). Animals were euthanatized with CO_2_ between 10:00 and 11:00 am on the morning of dissection. The liver was quickly removed, frozen in liquid nitrogen and stored at −80 °C. Total RNA from 12 animals (3 biological replicates in each of four diet-genotype comparisons) was isolated from liver using TRI Reagent (Molecular Research Center, Inc., Cincinnati, OH, USA), and assessed for quality in an Agilent Bioanalyzer 2100 (Agilent Technologies, Santa Clara, CA, USA). One μg of total RNA was used to transcribe DIG-labeled cRNA using AB Chemiluminescent RT-IVT Kit v2.0. Hybridization of 10 μg fragmented DIG-labeled cRNA to AB Mouse Genome Survey microarray (version 2.0), processing, chemiluminescence detection, imaging, auto-gridding, and image analysis were performed according to AB protocols, using the 1700 Chemiluminescent Microarray Analyzer Software v. 1.0.3. The AB Expression system software V2.0 was used to extract assay signal and signal-to-noise ratios from the images as previously described [17].

### Microarray data analysis

Signal intensities across microarrays were log transformed (base 2) and quantile normalized using the BRB-Array Tools software [18]. Statistical significance of the differentially expressed (DE) genes was ascertained via a regularized *t*-test based on the Bayesian statistical framework [19]. The magnitude of gene over-expression or under-expression was quantified by the difference in the log_2_ average signals between the treatment and control groups. Using analysis of variance, gene expression profiles in liver were analyzed for main effects involving diet and genotype, as well as diet by genotype interactions [18]. The MIAME guidelines compliant microarray data have been deposited in the National Center for Biotechnology Information (NCBI) Gene Expression Omnibus (GEO) repository, under accession number GSE35180.

### Ingenuity pathway analysis

Over-representation analysis (ORA) of canonical pathways was carried out by subjecting a pre-filtered list of differentially expressed genes with *P* < 0.01 and absolute fold-change ≥1.5-fold to Ingenuity Pathway Analysis (IPA). The Ingenuity Knowledge Base was used as the source for the reference genes. Fisher’s exact test p-values were used to estimate the significance of over-representation and corrections for multiple testing were made using the Benjamini-Hochberg procedure for false discovery rate [20]. Significantly enriched pathways were compared across treatments by 2-way clustering and then color coded according to the negative logarithm of their Fisher’s exact *P*-value.

IPA was further utilized to identify ‘upstream regulators’ whose activation/inhibition could explain the observed gene expression results. An overrepresentation analysis (Fisher’s exact test) was first performed to determine whether a regulator was enriched for differential expression of its target genes (the list of regulators and their target genes were obtained from the Ingenuity Knowledge Base). The overall activation/inhibition status of the regulator was inferred from the degree of consistency (up- or down-regulation) in the expression patterns of its target genes, expressed as a z-score. Regulators with z ≥ 2 or z ≤ −2 were considered to be activated or inhibited, respectively. A new score (C), combining the magnitude of the z-score with significance of overlap (*P*-value), was created according to the relationship: C = z-score * -logP. The C score was used to summarize the predicted effects from the upstream regulators.

### Quantitative real-time PCR

Complementary DNA (cDNA) was obtained by reverse transcription (SuperScript III First-Strand Synthesis System for RT-PCR, Invitrogen, Carlsbad, CA, USA) of 4 μg of RNA from each liver sample. cDNA was subjected to QIAquick PCR purification columns (Qiagen, Valencia, CA, USA). For qRT-PCR, samples from 6–12 individuals were tested for the *Acads−/−* and *Acads+/+* strains, respectively, using the microarray samples (3 per strain and condition) and additional samples collected under the same conditions.

Primers for amplification were designed using gene sequences obtained from the National Center for Biotechnology Information (NCBI) and Primer Express Software v3.0 (Life Technologies). Primers were synthesized by IDT (Integrated DNA Technologies, Coralville, IA, USA). The locations and sequences of primers are listed in Additional file 2: Table S2.

Gene expression levels were measured using the ABI PRISM 7900HT Sequence Detection System. Individual samples were run in triplicate. As an endogenous reference for normalization, we used the measurement of Cyclophilin (*Ppia*) cDNA in the same sample; *Ppia* levels were unaffected by diet and genotype on the arrays. Analysis was performed using SDS Software v2.3 for the 7900HT (Life Technologies). Relative quantification was calculated using the comparative C_T_ method (AB: User Bulletin #2: Relative quantification of gene expression. P/N 4303859 [21]. A two-tailed Student’s *t*-test was performed on ΔC_T_ values to evaluate strain differences in gene expression. *P* values of ≤ 0.05 were considered statistically significant.

## Results/Discussion

We investigated the effects of SCAD deficiency and high-fat diet in the liver. Our approach included analyses of mitochondrial oxidative function and nutrient-sensing signaling mechanisms, metabolic profiling of acylcarnitine levels and genome-wide transcriptional profiling. In each case, tissue was harvested from *Acads−/−* or *Acads+/+* mice only 2 days after initiating a single, high- or low-fat diet, to coincide with the time point at which the *Acads* mutants begin to eat less fat in a macronutrient diet selection paradigm [1]. Our overall aim was to uncover hepatic processes affected by fuel oxidation and ATP generation [3] that potentially could be linked to feeding behavior [17].

### *Acad*s genotype and HF diet increase acylcarnitine levels in the liver

**Fig. 1:**
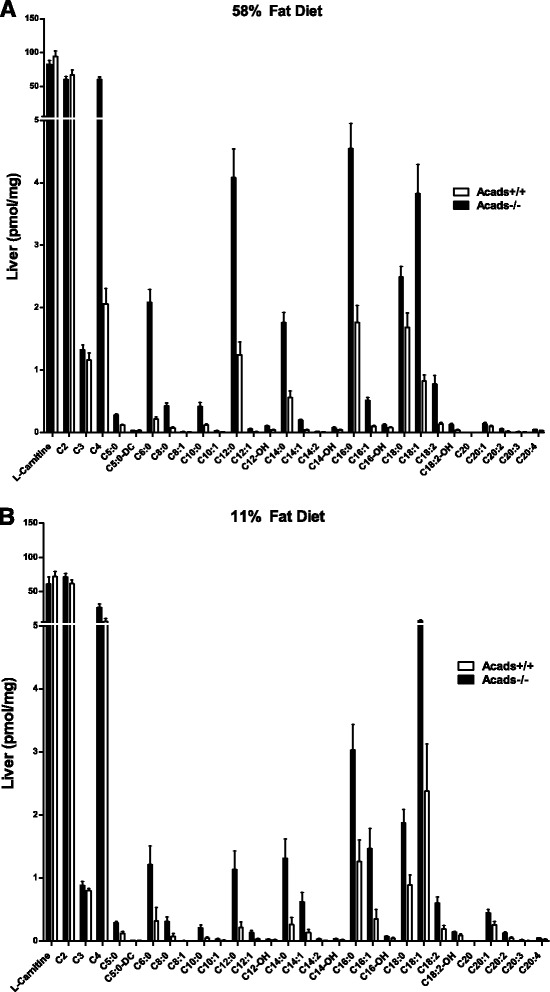
Effects of genotype and dietary fat level on liver acylcarnitine levels. Legend: Acylcarnitines were measured by tandem mass-spectrometry in liver tissue from Acads−/− and Acads+/+ fed high- (**a**) or low-fat (**b**) diet for 2 days. Values are means (pmol/mg protein) ± SEM. *n* = 6 animals per group. See Additional file 3: Table S3 for a summary of ANOVA results

Acylcarnitines are intermediary metabolites derived from mitochondrial acyl-CoA metabolism. To examine the biochemical effects of SCAD deficiency and dietary fat on lipid substrate metabolism in the liver, we measured acylcarnitines of *Acads−/−* and *Acads+/+* mice fed either HF or LF fat diet for 2 days. The analysis of variance (ANOVA) revealed significant main effects of diet on twenty (20) acylcarnitines, and of genotype on twenty-five (25) acylcarnitines (Additional file 3: Table S3). For example, on HF diet, the liver content of C4 (butyryl) and C5 (isovaleryl) was 30-fold (*P* < 0.0001) and 2-fold (*P* < 0.0001) higher, respectively, in *Acads−/−* compared with *Acads+/+* mice (Fig. 1), demonstrating the metabolic block. On LF diet however, the liver C4 content (*P* < 0.01) was only 4-fold higher in mutant animals. Six acylcarnitine species showed a significant diet-by-genotype interaction (see Additional file 3: Table S3). The accumulation of excess medium-chain and long-chain lipid byproducts, e.g., 3-fold higher levels of C12:0 and C14:0 acylcarnitines in HF-fed *Acads−/−* compared to HF-fed *Acads+/+* could have resulted, in part, from the enrichment of medium chain fatty acids from coconut oil in the HF diet. In liver, 20–25 acylcarnitine species were affected by genotype or diet, compared to only 5 species in plasma, as shown previously [17], suggesting a dysregulation of hepatic mitochondrial beta oxidation.

### *Acad*s genotype and HF diet increase pAMPK in the liver

**Fig. 2:**
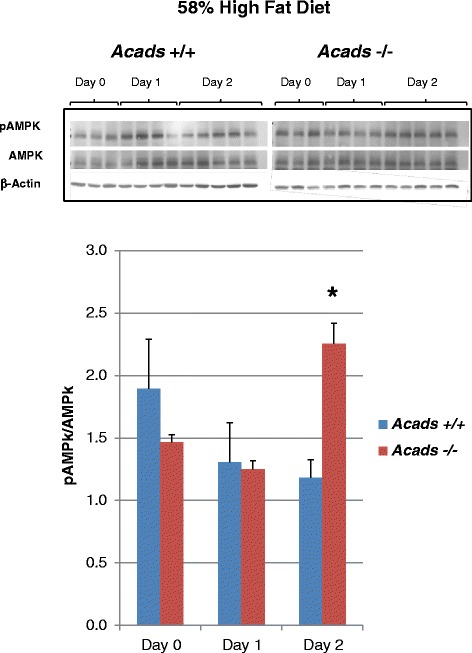
Effect of *Acads* genotype on pAMPK in the liver of mice fed high-fat diet. Legend: On chow (day 0) and after one day of high-fat diet, there was no effect of genotype on pAMPK level in liver. However, on day 2 of high-fat diet, pAMPK level was increased by ~40 % in *Acads−/−* compared to *Acads+/+* mice. Food was removed at 0700, animals were euthanized and tissues were harvested at 0900. Immunoblots for total AMPK, pAMPK and beta actin are shown. *n* = 3–5 per genotype and day. *P < 0.005, genotype comparison

Phosphorylation of AMP-kinase (pAMPK) is a key signal in the response to changes in energy balance including consumption of HF diet [22]. Cellular conditions that inhibit ATP production will result in a change in the AMP:ATP ratio and thus activate the AMPK signaling pathway. We therefore examined the effects of SCAD deficiency on hepatic pAMPK in *Acads*−/− mice fed HF diet for 2 d. Immunoblot analysis of liver tissue showed enhanced pAMP-kinase, compared to wild type controls, as evidenced by the ~40 % increase in (Thr-172) phosphorylation of the AMPK protein (Fig. 2). The adenosine monophosphate (AMP)-activated protein kinase (AMPK) phosphorylates numerous intracellular proteins and alters the transcription of genes involved in the regulation of energy metabolism including the transcriptional co-activation of PPAR-alpha. The net effect of AMPK activation is to turn off biosynthetic pathways such as lipogenesis and to activate catabolic pathways that produce ATP (e.g., fatty acid oxidation) [23]. This finding is consistent with a cellular “switch” from energy storage to energy release/use, suggesting that ATP availability is reduced under the conditions of impaired short chain fatty acid oxidation and high-fat diet, and agrees with our previous observation of AMPK activation in the hypothalamus of high-fat fed *Acads−/−* animals [17]. Finally, in light of previous reports that SCAD-deficient (also known as *Acads−/−*) mice are prone to fasting induced hypoglycemia [8], the animals in this experiment were tested a few hours after their most recent meal, when plasma glucose concentrations were similar between genotypes (data not shown). The absence of hypoglycemia in this study suggests that the observed AMPK activation in liver results from the metabolic effects of impaired short-chain fatty acid catabolism.

### *Acads* inactivation inhibits oxygen consumption rate of isolated liver mitochondria in a substrate-specific manner

We found that pAMPK was significantly elevated in the liver of HF-fed, *Acads−/−* mice, suggesting a depletion of ATP. Mitochondria are the main sites of cellular ATP production from substrate oxidative phosphorylation [24] and play a critical role in fatty acid oxidation. Thus, we investigated the effects of SCAD deficiency on mitochondrial respiration in isolated hepatic mitochondria from *Acads−/−* mice fed HF diet. Because the SCAD protein is the first enzyme involved in the short-chain fatty acid beta oxidation spiral, we tested mitochondria for their ability to oxidize exogenously added fatty acids.

#### *Acads−/−* mitochondria function normally

**Fig. 3:**
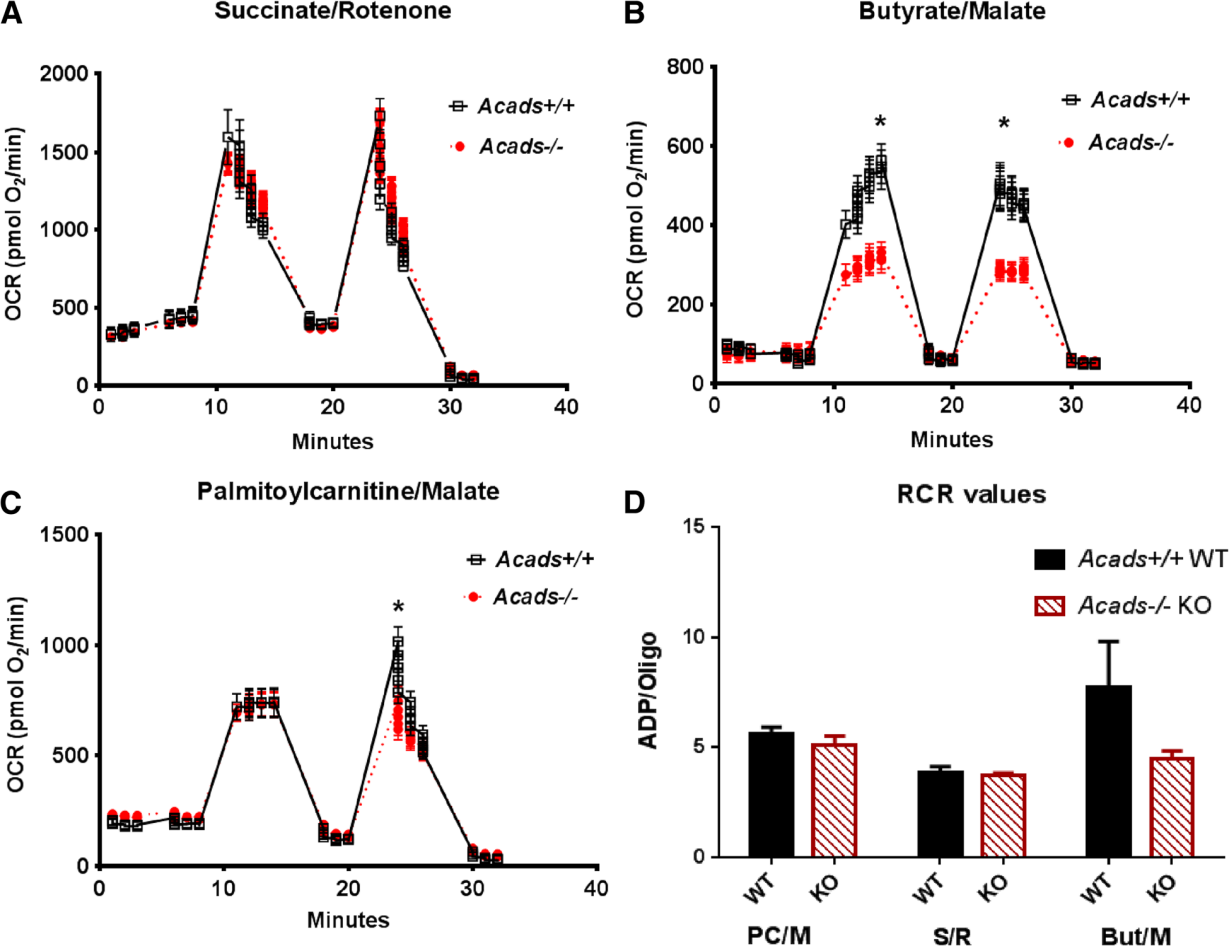
Mitochondrial oxygen consumption rate. Legend: Mitochondrial oxygen consumption rate in isolated liver mitochondria in the presence of various substrates. Experiments were run on a SeaHorse XF24 extracellular flux analyzer to obtain bioenergetics profiles from *Acads−/−* and *+/+* mice. OCR, oxygen consumption rate. Data are displayed as absolute, point-to-point oxygen consumption rates (pmol/min/well). **a** OCR in the presence of succinate/rotenone. **b** OCR with butyrate/malate. **c** OCR with palmitoylcarnitine/malate. **d** respiratory control ratio (RCR); calculated as the state 3 respiratory rate divided by the state 4 respiratory rate, obtained from the ADP-linked/oligomycin OCR readings. Samples were run in triplicate. Data represent means ± SE for *n* = 3-9 animals. *P < 0.05, *Acads−/−* vs. *Acads+/+*

To test the hypothesis of differences in mitochondrial efficiency between *Acads*−/− and *Acads+/+* mice, we measured real-time oxygen consumption rates (OCR) in isolated liver mitochondria using an extra-organelle flux assay. OCR was measured in the presence (state 3) or the absence (state 4) of ADP, along with the ratio of these two rates---the respiratory control ratio (RCR) [25]. We first tested the function of complex II (a.k.a. succinate dehydrogenase), which is an enzyme complex bound to the inner mitochondrial membrane that participates in both the citric acid cycle and the electron transport chain. The results showed that in the presence of succinate + rotenone (complex II), basal respiration, leak respiration, uncoupled respiration and non-mitochondrial respiration were similar between mitochondria from *Acads−/−* and wild-type mice (Fig. 3a). Additionally, the RCR values indicate that the mitochondria used in these experiments were intact and functional; there were no differences between genotype groups (Fig. 3d), demonstrating that SCAD deficiency does not result in systemic mitochondrial dysfunction.

#### *Acads−/−* mitochondria have impaired ability to oxidize butyrate

Mitochondria isolated from the liver of *Acads*−/− mice were also tested for their ability to respire on a short chain lipid using butyrate combined with the anaplerotic substrate malate. We found that both ADP-stimulated and uncoupled respiration were significantly reduced in *Acads−/−* mitochondria compared with *Acads+/+* (Fig. 3b). However, neither basal nor leak (oligomycin inhibited) respiration were significantly different between genotypes. These changes resulted in a slight, although not statistically significant, decrease in the RCR obtained from *Acads−/−* mitochondria (Fig. 3d), suggesting the possibility of a substrate-specific impairment in *Acads−/−* mitochondrial function when using butyrate/malate. Importantly, the *Acads−/−* mitochondria retained their capacity to respond to both ADP and an uncoupling agent, although at lower rates than wild-type mitochondria. This result is consistent with the different but overlapping substrate-chain-length specificities of acyl-CoA dehydrogenases, e.g., that medium chain acyl-CoA dehydrogenase (MCAD) may be active toward some esters of short-chain fatty acids such as C6, but not toward those of long-chain fatty acids [26]. To examine more fully the implications of SCAD deficiency on energy production, we then measured mitochondrial respiration using long-chain fatty acids as the substrate.

#### Maximal, but not ADP-linked, long chain fatty acid respiration was reduced by SCAD deficiency

We measured OCR in liver mitochondria from *Acads*−/− mice using palmitoylcarnitine/malate as substrates and found no difference in basal, resting or ADP-stimulated respiration (Fig. 3c), or in RCR (Fig. 3d), compared to controls. The absence of a significant difference in palmitoylcarnitine OCR under these conditions is somewhat surprising but may indicate that partial oxidation of long-chain fatty acids is sufficient to meet the energy demands of the cell under most conditions. When mitochondria from wild type liver were fed fatty acids as substrate, respiratory control by the phosphorylation of ADP acted to limit activity of the electron transport chain. Then, when the chemical uncoupler FCCP was applied and electron transfer was no longer controlled by the proton gradient, we observed an increase in oxygen consumption that was 39 % greater than ADP-stimulated respiration, providing evidence for enhanced mitochondrial spare capacity under conditions of heightened energy demand. However in isolated mitochondria from *Acads−/−* liver, maximal uncoupled respiration merely reached levels similar to those observed in ADP-stimulated respiration, indicating a nearly complete decrement in spare respiratory capacity when using a traditional long chain fatty acid substrate. Therefore, it appears that under normal circumstances the long and medium chain acyl-CoA dehydrogenases can supply sufficient reducing equivalents to the electron transport chain [27]. This residual activity may help explain the reports of weaker clinical symptoms for SCAD deficiency than for VLCAD deficiency [28, 29]. Nevertheless, in the absence of functional SCAD enzyme, a decrement in the beta-oxidation of C4-C6 fatty acids may exceed the mitochondrial capacity for energy production under conditions of greater energy demand, such as that induced by FCCP, supporting our finding that mitochondrial oxidative phosphorylation is impaired in isolated hepatic mitochondria from *Acads−/−* animals.

#### Mitochondrial copy number

The induction of AMPK by energy restriction is known to activate the transcription coactivator peroxisome proliferator-activated receptor gamma, coactivator 1 alpha (PPARGC1A, also known as PGC1-a) and to enhance mitochondrial biogenesis [23]. Additionally, prolonged high-fat diet intake has been shown to induce changes in mtDNA content [30]. Here we measured the hepatic mtDNA content of *Acads−/−* and *Acads+/+* mice using qRT-PCR and found no significant differences in mtDNA content (Additional file 4: Figure S1), in mice that were fed HF diet for 2 days. Thus we can exclude the possibility that differential mitochondrial proliferation is responsible for the observed genotype difference in mitochondrial OCR.

### *Acads* genotype and high-fat diet alter gene expression in the liver

Energy balance is maintained by changes in feeding behavior and metabolism which can be regulated by gene expression. To identify the transcriptional responses to *Acads* inactivation and dietary fat, we used microarrays to profile the expression of 32,381 transcripts in the liver of *Acads−/−* and *Acads+/+* mice fed either high- or low-fat diet. We found more pronounced differences in transcript abundance (absolute fold-change ≥ 1.5 and nominal *P*-value of <0.01) between the two diet comparisons than between the two genotype comparisons (Table 1).

**Table 1:** Summary of differentially expressed genes

Experimental comparison	Total	Increased	Decreased
HF: *Acads−/−* vs. *Acads+/+*	182	108	74
LF: *Acads−/−* vs. *Acads+/+*	72	31	41
*Acads+/+*: HF vs. LF	284	97	187
*Acads−/−*: HF vs. LF	287	145	142

The Applied Biosystems Mouse Genome Survey Microarray v2.0 was used to identify in liver the differential expression of genes by genotype or diet. A total of 12 arrays were used. Lists of differentially expressed genes for each comparison are based on a fold change of ≥1.5 and a *P* value of <0.05

### *Acads* genotype alters hepatic gene expression

**Fig. 4:**
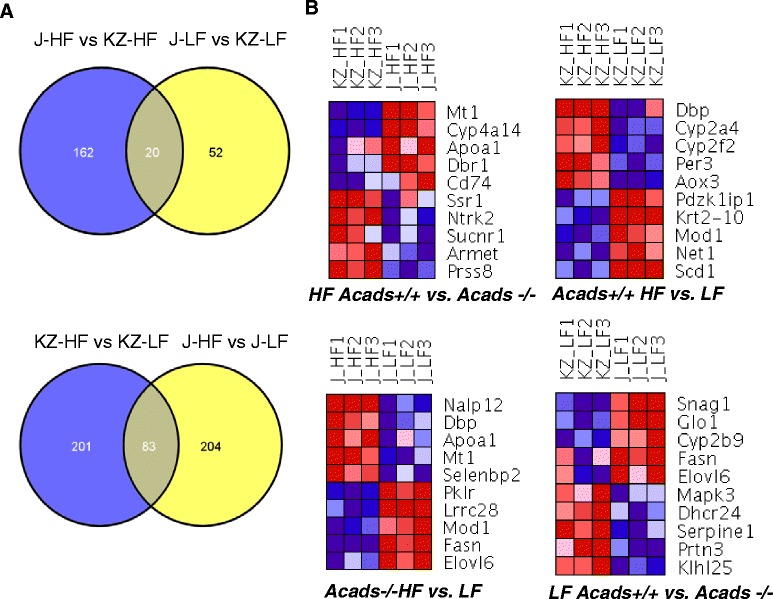
Venn diagrams of differentially expressed genes affected by diet and genotype along with two-way Ward clustering of top 5 increased and top 5 decreased genes. Legend: **a** Overlap among significantly differentially expressed (DE) genes from each treatment (genotype, diet). Genes with an absolute fold change ≥ 1.5-fold and a nominal p-value <0.01 were considered to be significantly DE. **b** Two-way Ward clustering of top significantly DE genes. Treatments are organized by columns and genes by rows (expression levels in the heatmap are color-coded with blue representing lower and red representing higher levels of mRNA abundance). J, BALB/cByJ or *Acads−/−*; KZ, BALB/ByKZ, or *Acads+/+*

Microarray analysis of gene expression in the liver, under the HF diet condition, revealed changes in 182 genes due to genotype, of which 108 genes showed increased levels in *Acads*−/− mice (Table 1). LF diet resulted in a considerably weaker transcriptional response, as only 72 genes were differentially expressed, with 31 increased in *Acads*−/− mice. Of these, 20 were common to both the HF and LF diets, with an equal number of up- and down-regulated transcripts (Fig. 4a and b). The extent of this overlap was statistically significant, based on the hypergeometric test (*p* < 2.92e-13). For example, we uncovered the genotype-regulated induction of *Glo1*, encoding glyoxalase 1, with both the HF and LF diets, by a factor of 2.0 and 2.5, respectively. The glyoxalase system acts to degrade endogenous reactive dicarbonyls such as glyoxal formed by lipid peroxidation and/or methylglyoxal derived from glycolysis [31]. Glyoxal and methylglyoxal are toxic byproducts of metabolism, which if not neutralized, can produce deleterious modifications of protein and DNA through glycation [32]. Activation of the hepatic glyoxalase pathway in *Acads* mutants, compared to wild type controls, suggests an enzymatic response to the accumulation of intermediates brought about by SCAD deficiency. The observed *Glo1* induction in this model is consistent with the inferred activation of *Nfe2l2/Nrf2* (see “Predicted Upstream Regulators” below), an oxidative stress response gene and transcriptional regulator of *Glo1* [32]. A more detailed list of the top 25 up- and down-regulated genes for each of the above treatments is provided in Additional file 5: Table S4.

### High-fat diet alters hepatic gene expression

Two hundred and eighty-seven (287) diet-responsive genes were differentially expressed in *Acads*−/− mice, with 142 of them decreased in the HF fed group. By contrast in wild type controls, two hundred and eighty-four genes (284) were differentially expressed, with 187 genes down-regulated in the HF fed group. Eighty-three (83) genes were altered by high-fat diet in both *Acads−/−* and *Acads+/+* mice (Fig. 4a) and of these, 50 were down-regulated. The overlap in these 83 genes was highly significant by the hypergeometric test (*P* < 9.87e-12). Diet-modified genes involved in fatty acid (FA) synthesis and elongation were strongly decreased by HF (vs. LF) diet in both mouse strains, usually with greater fold changes noted in *Acads−/−* animals. For example, *Me1* (malic enzyme; also known as *Mod1*) was decreased by 5.8-fold in *Acads−/−* and by 3.5-fold in *Acads+/+* mice. *Me1* induction by carbohydrate consumption in the LF diet groups, independent of genotype, may be responsible for this outcome (Fig. 4b), producing enzymatic action that links the glycolytic and citric acid cycles. As another example, *Elovl6* (elongation of very long chain fatty acids) was reduced by 25-fold in *Acads−/−* but only by 3.3-fold in wild type controls, suggesting a consequence of impaired SCFA oxidation in the mutants.

### Genotype x diet interaction effects

A total of 137 genes demonstrated significant diet x genotype interactions at the *P* < 0.01 level (Additional file 6: Table S5). For nearly all of these transcripts, the interaction model explained substantially more of the variation in expression than did a model based only on main effects. Twenty-nine (29) of these transcripts encode for mitochondrial proteins, e.g., *Acsl3* (activates LCFAs for both synthesis and beta oxidation), *Cyp27a1* (drug metabolism, cholesterol synthesis), *Gpx1* (glutathione peroxidase), *Ndufc2* (complex I mitochondrial enzyme), *Uqcrfs1* (complex III), as well as three cytochrome c oxidase (*Cox*) components of complex IV: *Cox5b*, *Cox7c* and *Cox11*. Notably, genes in three complexes of the electron transport chain were affected by a genotype x diet interaction, and of these, *Ndufc2*, *Uqcrfs1*, and *Cox11* were more responsive to genotype. The possibility that these specific results may reflect a secondary defect in oxidative phosphorylation, and thus ATP production, in the *Acads−/−* mice [33], remains speculative.

### Biological pathways are altered by *Acads* and dietary fat level

**Fig. 5:**
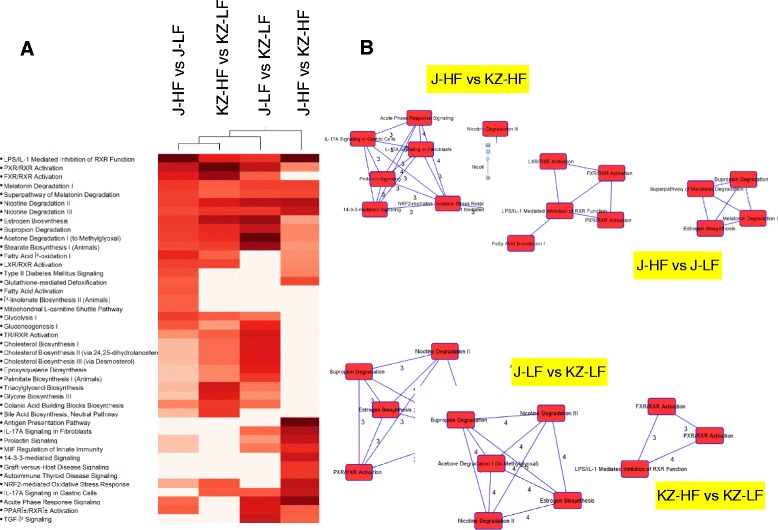
Enrichment of canonical pathways for diet- and genotype-specific comparisons. Legend: Analysis was conducted using the Ingenuity Pathway Analysis tool, based on the Ingenuity Knowledge Base. **a** 2-way clustering based on pathway significance levels (Fisher’s exact test). Heat-map is color-coded from white to red according to an ascending scale of negative log_10_P-value. Pathways with a negative log_10_P ≥ 3 in at least one comparison are shown. **b** Sharing of common genes among top-ranking pathways in each comparison. Pathways sharing at least 3 genes for any pathway-pair are shown. The number of genes shared between any two pathways are also indicated. J, BALB/cByJ or *Acads−/−*; KZ, BALB/cByKZ or *Acads+/+*

Canonical pathway analysis using IPA highlighted key alterations associated with short term high-fat diet and/or genotype (Fig. 5a), e.g., a significant inhibition of fatty acid and triacylglycerol synthesis in both *Acads+/+* and *Acads−/−* liver. This process was reflected by the decreased expression of *Scd1, Fasn, Srebf1,* and *Mod1* (fatty acid synthesis), as well as *Gpam* and *Elovl6* (fatty acid storage and elongation). IPA analysis also found strong enrichment of “Fatty Acid Beta Oxidation” in HF-fed *Acads−/−* mice, e.g., genes involved in long-chain FA degradation (*Acsl3, Acsl5, Slc17a2, Slc17a5*) and in peroxisomal beta oxidation (see below).

In *Acads*−/− mice fed HF diet for 2 days, all three top enriched pathways involved *Rxra* (retinoid X receptor alpha), which forms heterodimers with other nuclear receptors such as PPARs [34], and is required for *Ppara* transcriptional activity on fatty acid oxidation genes [34, 35]. A potential mechanism for the induction of RXRA/PPARA pathways is butyryl CoA buildup in the cell [36], which is expected to occur with SCAD deficiency. Additionally, the transcriptional regulation of other genes is consistent with activation of alternate lipid handling pathways in the liver, including both beta- (mitochondria, peroxisomes) and omega-oxidation (microsomes). Long-chain fatty acids (C14-C20) are catabolized by either mitochondrial or peroxisomal beta-oxidation pathways, whereas very long-chain fatty acids (C22) are chain-shortened through peroxisomal beta-oxidation before being completely oxidized in the mitochondria [37]. The increased expression of *Acaa1a, Ehhadh* and *Decr2,* genes encoding key enzymes of peroxisomal beta-oxidation [38], provides evidence that peroxisomal oxidation was enhanced in *Acads−/−* mice, but not in wild type. Further support for alternate lipid handling was indicated by the 3-fold induction of *Acot1* (acyl-CoA thioesterase 1) which predominantly hydrolyzes long-chain (C12-C16) acyl-CoA molecules. Evidence for an adaptive metabolic response was also observed in the 5 to 6-fold induction in *Cyp4a14* and *Cyp4a10* in *Acads−/−* liver by HF diet. The CYP4A subfamily of cytochrome P450 enzymes, located in the endoplasmic reticulum, function in microsomal omega oxidation, i.e., they metabolize medium and long chain length fatty acids at their omega-carbon atom to produce dicarboxylates as an alternative route to mitochondrial beta oxidation [39]. Normally, omega oxidation accounts for less than 10 % of total fatty acid oxidation in the liver [40], but when beta oxidation is defective, microsomal omega-oxidation may provide a means for eliminating toxic levels of free fatty acids [41]. For example, this pathway includes mitochondrial aldehyde dehydrogenase (*Aldh2*), encoding an antioxidant defense protein, which was induced by 5-fold in *Acads−/−* animals.

A comprehensive list of all the significantly enriched IPA pathways in each experimental comparison is provided in Additional file 7: Table S6.

### Predicted upstream regulators of gene expression

**Fig. 6:**
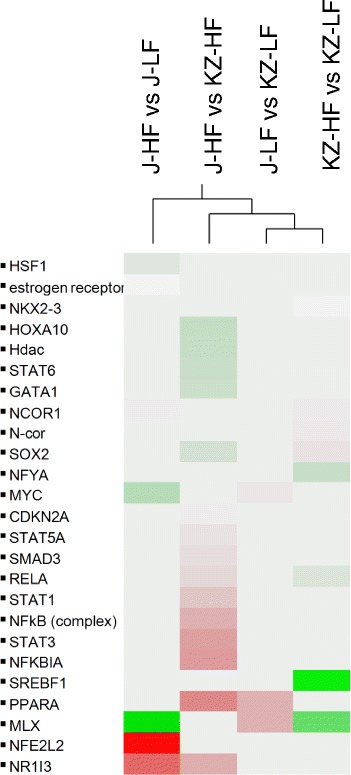
Clustering of candidate upstream activators. Legend: Average-linkage clustering of candidate upstream regulators identified as significantly activated or inhibited in at least one of the four treatments (|z| ≥ 2). Clustering was performed based on the *C* score and visualized by a heat-map. Predicted activations and inhibitions are shown in red and green, respectively, and shaded by the magnitude of the *C* score. J, BALB/cByJ or *Acads−/−*; KZ, BALB/cByKZ or *Acads+/+*

To better understand the possible molecular basis for the observed transcriptomic changes, we investigated the predicted activation or inhibition of upstream transcription factors (TF) with at least 5 target genes in the list of differentially expressed genes (absolute fold change ≥ 1.5 and nominal p-value of <0.01) for a given diet or genotype comparison. In the diet comparison involving *Acads−/−* mice, we noted the significant, predicted activation of *Nfe2l2* (nuclear factor, erythroid derived 2, like 2) as well as the significant inhibition of *Mlx* (MAX-like protein X), a transcriptional activator of glycolytic target genes (Fig. 6). The predicted activation or inhibition status for the full list of upstream regulators (beyond TFs) is shown in Additional file 8: Table S7. The similarities and dissimilarities in upstream regulator activation or inhibition across the 4 treatment groups are shown via hierarchical clustering in Additional file 9: Figure S2.

**Fig. 7:**
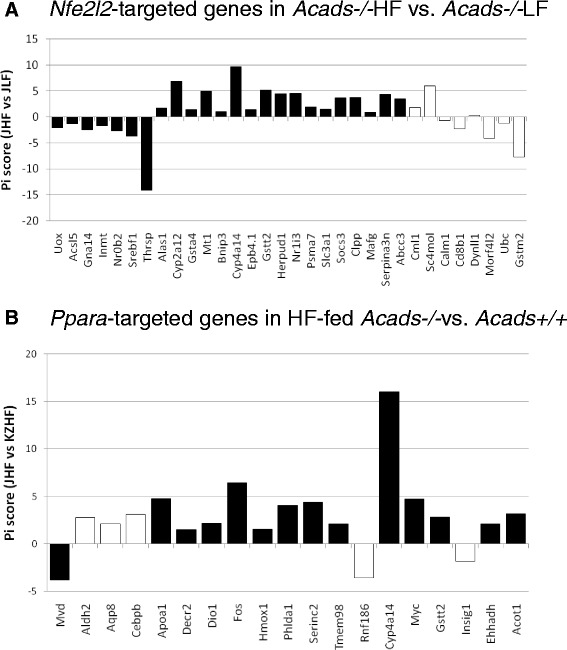
Predicted upstream regulators of gene expression in microarray. Legend: Upstream regulator analysis on gene expression in microarray was restricted to transcription factors with at least 5 target genes in the list of differentially expressed genes (absolute fold change ≥ 1.5 and nominal *P*-value ≤ 0.01) for a given diet or genotype comparison. **a** *Nfe2l2*-targeted genes in *Acads−/−* mutant mice fed high-fat (HF) vs. low-fat (LF) diet. **b** *Ppara-*targeted genes in HF-fed *Acads−/−* vs. *Acads+/+* mice. Pi = neglogP*logratio. Genes whose direction of expression was consistent with activation of *Nfe2l2* (**a**) or *Ppara* (**b**) are colored in black and those with inconsistent direction of expression are shown in white. J, BALB/cByJ or *Acads−/−*; KZ, BALB/cByKZ or *Acads+/+*

Using differentially expressed genes obtained from the diet comparison (HF vs. LF) in *Acads*−/− animals, upstream regulator analysis revealed 24 significantly differentially expressed genes whose direction of expression was consistent with activation of the transcription factor *Nfe2l2* (Fig. 7a). *Nfe2l2* (synonymous with *Nrf2*) binds to antioxidant response elements (ARE) in the upstream promoter region and initiates the transcription of a variety of cyto-protective target genes involved in the response to oxidative stress caused by xenobiotic exposure and other factors [42]. Several of these genes (e.g., *Gsta4*, *Cyp4a14*) are also known to regulate various aspects of lipid metabolism, e.g., the alpha class of glutathione S-transferases, e.g., *Gsta4,* functions to detoxify products of lipid peroxidation, as illustrated by significant enrichment of the “Glutathione-mediated Detoxification” pathway, when both SCAD deficiency and HF diet are involved (Fig. 5a). As well, dysregulation of the cytochrome P450 (*Cyp*) 4a subfamily of genes by HF diet may be related to the demands for transformation of excess fatty acids which are substrates for *Cyp4* isoforms. Overall, the inferred activation of *Nfe2l2*/ARE and the explicit induction of the *Cyp4a* subfamily in *Acads−/−* liver, appear to be directed at processing the incomplete products of mitochondrial beta oxidation [43]. Confirmation of this result will require a direct test of NFE2L2 activity or studies using *Nfe2l2−/−* mice.

Upstream regulator analysis also was performed on differentially expressed transcripts obtained by comparing genotypes (*Acads−/−* vs. *Acads+/+*) in HF-fed animals. Fourteen (14) gene targets were identified whose direction of expression suggested PPARA activation, including *Cyp4a14* whose mRNA was elevated by nearly 9-fold (Fig. 7b). The predicted target genes represent the diverse functions of *Ppara,* including lipoprotein metabolism (*Apoa1)*, microsomal omega oxidation (*Cyp4a10*, *Cyp4a14*), peroxisomal beta oxidation (*Ehhadh*), hydrolysis of fatty acyl-CoAs by thioesterases (*Acot1*), and biotransformation (*Gstt2*). Overall, these effects are consistent with the regulatory role of PPAR-alpha in energy metabolism, including all three hepatic fatty acid oxidation systems, i.e., mitochondrial and peroxisomal beta oxidation, as well as microsomal omega oxidation in the endoplasmic reticulum [38].

Both chronic high-fat feeding and fasting activate PPARA signaling in mouse liver, as part of the physiological response to an increased need for fatty acid oxidation [44]. In our short-term feeding experiment, the inferred PPARA activation may have been triggered by accumulated acylcarnitines, both in the circulation [17] and in liver tissue [45]. This result follows the pattern of PPARA induction reported previously for both the *Lcad−/−* and *Vlcad−/−* mouse strains with chow feeding [46]. It is also possible that PPARA signaling and fatty acid oxidation pathways were activated by a low energy state, as indicated by the enhanced hepatic pAMPK (Fig. 2) and reduced mitochondrial OCR in HF-fed *Acads−/−* mice (Fig. 3b).

### Quantitative RT-PCR validation of gene expression in liver

To investigate the potential evidence for RXR-alpha activation and determine the effects of high-fat diet and SCAD deficiency on common partners of *Rxr*, we used quantitative RT-PCR to measure the expression of eleven nuclear receptor genes, as well as some glucose metabolism genes, in all four diet and genotype comparisons (total of 44 tests) (Table 2). Statistical significance for differential expression in microarray analysis was confirmed in 36/44 or 82 % of the tests. In addition to this validation study, we selected a set of 27 transcripts to represent metabolic processes and examined their expression as a function of diet in *Acads−/−* mice. The results showed significant (*P* < 0.05) differences between high- and low-fat diets in 23/27 or 85 % of the genes (Table 3). We observed 100 % agreement in the direction of expression changes between the two platforms, and thus have confidence that the qRT-PCR and microarray results are congruent, and that they reveal a predominant effect of diet, but not of genotype.

**Table 2:** Gene expression analyses by qRT-PCR for diet and genotype comparisons

	Diet comparisons					Genotype comparisons			
Gene Symbol	Microarray		qRT-PCR		Gene Symbol	Microarray		qRT-PCR	
	FC	*p*-value	FC	*p*-value		FC	*p*-value	FC	*p*-value
*Acads−/−* HF vs. LF					HF *Acads−/−* vs. *Acads+/+*				
Acacb	ND	ND	−2.86	0.001	Acacb	4.02	0.230	1.14	0.652
G6pc	−3.20	0.000	−2.94	0.000	G6pc	ND	ND	−1.07	0.606
Nr0b2 (SHP-1)	−1.87	0.000	1.40	0.063	Nr0b2	ND	ND	1.33	0.253
Nr1h3 (LXR)	ND	ND	1.15	0.234	Nr1h3	ND	ND	−1.08	0.512
Nr1h4 (FXR)	ND	ND	−1.10	0.619	Nr1h4	ND	ND	1.22	0.308
Nr1i2 (PXR)	ND	ND	−1.31	0.081	Nr1i2	ND	ND	−1.38	0.024
Nr1i3 (CAR)	2.40	0.000	4.23	0.001	Nr1i3	ND	ND	1.43	0.161
Pklr	−5.28	0.000	−3.93	0.000	Pklr	ND	ND	1.18	0.372
Rxra	2.28	0.000	1.56	0.004	Rxra	ND	ND	1.00	0.981
Srebf1	−2.03	0.000	−1.61	0.095	Srebf1	ND	ND	−1.04	0.829
Sult1a1(Phase II DME)	2.55	0.000	3.28	0.000	Sult1a1	ND	ND	1.40	0.666
*Acads+/+* HF vs. LF					LF *Acads−/−* vs. *Acads+/+*				
Acacb	−5.13	0.000	−2.99	0.003	Acacb	ND	ND	1.09	0.704
G6pc	ND	ND	−1.67	0.001	G6pc	ND	ND	1.64	0.000
Nr0b2 (SHP-1)	ND	ND	−1.12	0.719	Nr0b2	ND	ND	−1.18	.551
Nr1h3 (LXR)	ND	ND	1.07	0.757	Nr1h3	ND	ND	−1.17	0.457
Nr1h4 (FXR)	ND	ND	1.16	0.568	Nr1h4	ND	ND	1.56	0.125
Nr1i2 (PXR)	ND	ND	1.32	0.197	Nr1i2	ND	ND	1.25	0.311
Nr1i3 (CAR)	1.87	0.000	2.60	0.002	Nr1i3	ND	ND	−1.14	0.688
Pklr	−3.56	0.000	−3.65	0.000	Pklr	ND	ND	1.27	0.188
Rxra	ND	ND	1.31	0.034	Rxra	ND	ND	−1.19	0.147
Srebf1	−1.71	0.009	−1.56	0.070	Srebf1	ND	ND	−1.01	0.974
Sult1a1(Phase II DME)	ND	ND	1.67	0.009	Sult1a1	ND	ND	−1.41	0.046

Representative genes were selected to validate the microarray results in liver. Overall, we observed concordant changes in the array and the qPCR. With the diet comparisons alone, 12 PCR assays reached statistical significance in a two-tailed Student’s *t*-test performed on ΔCt values

*ND,* not detected

**Table 3:** Gene expression analyses by qRT-PCR for diet comparison in *Acads−/−* mice

		Microarray		qRT-PCR	
Gene Symbol	Gene Name	Fold change	p-value	Fold change	p-value
Aacs	acetoacetyl-CoA synthetase	−3.34	0.000	−3.34	0.001
Abcc3	ATP-binding cassette, sub-family C (CFTR/MRP), member 3	1.98	0.004	2.07	0.003
Acaa1a	acetyl-Coenzyme A acyltransferase 1A	1.82	0.003	1.59	0.041
Acot1	acyl-CoA thioesterase 1	3.11	0.039	4.15	0.011
Apol9b	apolipoprotein L 9b	3.00	0.022	2.34	0.000
Decr2	2-4-dienoyl-Coenzyme A reductase 2, peroxisomal	2.04	0.003	2.04	0.011
Dhrs13	dehydrogenase/reductase (SDR family) member 13	1.82	0.046	1.42	0.018
Ehhadh	enoyl-Coenzyme A, hydratase/3-hydroxyacyl Coenzyme A dehydrogenase	1.64	0.025	1.93	0.014
Elovl6	ELOVL family member 6, elongation of long chain fatty acids (yeast)	−24.79	0.021	−11.11	0.000
Fasn	fatty acid synthase	−7.21	0.000	−6.69	0.000
Gpam	glycerol-3-phosphate acyltransferase, mitochondrial	−5.70	0.000	−2.26	0.000
Gpi1	glucose phosphate isomerase 1	−2.49	0.001	−2.16	0.000
Gpr146	G protein-coupled receptor 146	2.03	0.003	2.17	0.000
Gstm2	glutathione S-transferase, mu 2	−2.33	0.002	−1.76	0.000
Macrod2	MACRO domain containing 2	−1.97	0.005	−1.11	0.444
Mod1/Me1	malic enzyme 1, NADP(+)-dependent, cytosolic	−5.81	0.025	−5.35	0.000
Nars	asparaginyl-tRNA synthetase	−1.65	0.025	−1.49	0.133
Pdhb	pyruvate dehydrogenase (lipoamide) beta	−1.83	0.015	−1.58	0.007
Pdk1	pyruvate dehydrogenase kinase, isoenzyme 1	−1.72	0.004	−1.54	0.307
Pdk4	pyruvate dehydrogenase kinase, isoenzyme 4	−3.98	0.018	−2.00	0.004
Pklr	pyruvate kinase liver and red blood cell	−5.28	0.000	−5.28	0.000
Rps6kl1	ribosomal protein S6 kinase-like 1	2.61	0.015	1.63	0.026
Scd1	stearoyl-Coenzyme A desaturase 1	−8.57	0.024	−19.17	0.000
Slc17a4	solute carrier family 17 (sodium phosphate), member 4	−2.74	0.009	−1.91	0.000
Slc27a5	solute carrier family 27 (fatty acid transporter), member 5	1.62	0.004	1.77	0.000
Sucnr1	succinate receptor 1	−2.33	0.033	−2.55	0.379
Uox	urate oxidase	−1.65	0.015	−1.33	0.039

Representative genes were selected to validate the microarray results in liver, using real-time RT-PCR. For the comparison of *Acads*−/− HF diet vs. LF diet, 27 genes are shown. There was strong concordance between the two platforms, i.e., 23 PCR assays reached statistical significance in a two-tailed Student’s *t*-test performed on ΔCt values

### Summary of fatty acid and glucose metabolism genes altered by SCAD deficiency and high-fat diet

**Fig. 8:**
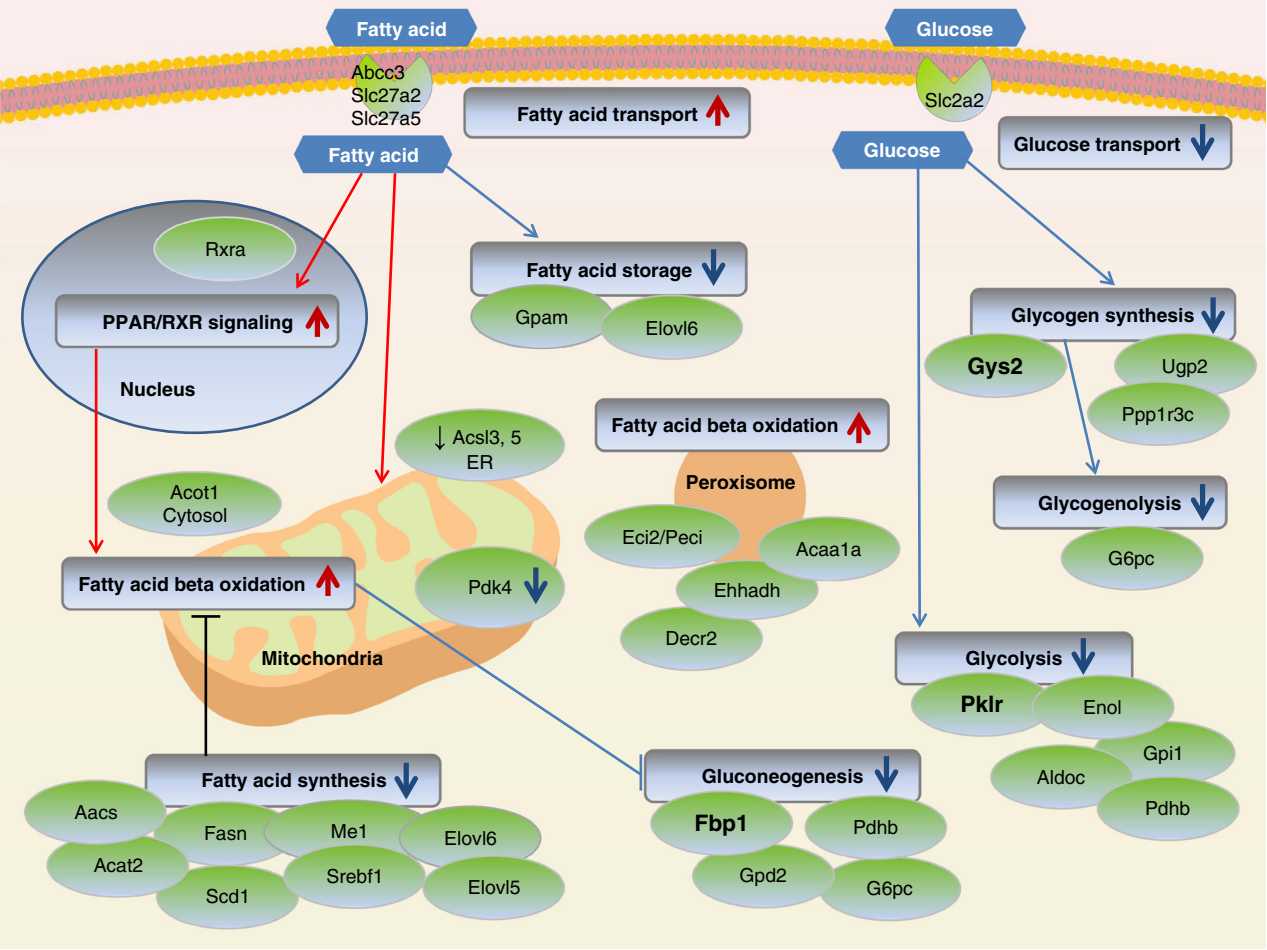
Overview of differentially expressed genes in fatty acid and glucose metabolism in the liver of *Acads*−/− mice fed high- or low-fat diet. Legend: The gene nodes and their interactions are presented after the manner of Zhang et al. [47], based on significantly differentially expressed genes from the diet comparison in *Acads−/−* mice. Large arrows indicate the direction of gene expression (red, increased; blue, decreased) for the gene nodes associated with each pathway

The transcriptional changes in hepatic fatty acid and glucose metabolism in *Acads−/−* mice fed HF or LF diet are summarized in Fig. 8, in a manner similar to that of Zhang et al. [47].

In general, fatty acids are catabolized in the mitochondria and/or peroxisomes to produce energy through beta oxidation while excess lipids are stored as triglycerides. We observed the increased expression of genes encoding enzymes involved in peroxisomal beta oxidation and in cytosolic hydrolysis of C12-C16 acyl-CoAs, as well as increased expression of genes involved in routing lipids for metabolism. These include FA transport genes (*Abcc3*, *Slc27a2*, *Slc27a5*) and peroxisomal *Eci2* (formerly *Peci*) localized in both mitochondria and peroxisomes. In addition, acyl-CoA synthetase gene expression was decreased (e.g., *Acsl5*), which may promote the induction of peroxisomes as an alternate route of lipid disposal [48]. The increased expression of *Rxra* indicates activation of the PPAR/RXR signaling pathway, as do the upstream regulator analyses, particularly the increased expression of PPARA-target gene *Cyp4a14* (Fig. 7b). The expression of genes encoding proteins involved in lipid biogenesis, as well fatty acid elongation and desaturation, was decreased in *Acads−/−* mice fed HF vs. LF diet, (Fig. 8).

The observed changes in gene expression are consistent with limited hepatic glucose utilization. For example, the expression of genes involved in glucose transport, as well as glycolysis and glycogen synthesis, including rate-limiting enzymes *Pklr* (−5.3-fold) and *Gys2*, respectively, was decreased. Furthermore, the expression of gluconeogenesis genes *G6pc* and *Gpd2*, together with rate-limiting enzyme fructose-1,6-bisphosphatase (*Fbp1)*, was much lower in *Acads−/−* liver. Notably, we also observed a ~4-fold reduction in mitochondrial *Pdk4*, validated by qRT-PCR, along with a decrease in *Pdk1* (Table 3), suggesting increased pyruvate dehydrogenase complex (PDC) activity. This result appears paradoxical, based on what is known about the transcriptional regulation of pyruvate dehydrogenase kinase, and the prediction of an up-regulation of *Pdk4* in response to increased fat supply from the HF diet [49]. However, the decreased *Pdk4* gene expression may reflect the short-term nature of diet exposure (2 days), possibly indicating that the metabolically active liver tissue has not yet “switched” on its PDC activity to maintain ATP levels, i.e., by regulating entry of glycolytic products into the tricarboxylic acid cycle. These results suggest that the altered fuel utilization in *Acads−/−* animals differs from that of the classic fed-fasted cycle [49].

## Conclusion

We identified altered acylcarnitine levels, enhanced pAMP-kinase signaling, and decreased mitochondrial oxidative function in the liver of *Acads−/−* animals fed high-fat diet for two days. Additionally, diet-induced liver transcriptomic responses in SCAD-deficient mice support increased RXR/PPARA signaling and upregulation of genes involved in multiple lipid handling pathways, including fatty acid oxidation. Nevertheless, the results of metabolite analyses suggest a substantial limitation in short-chain fatty acid oxidation, that may account for the activation of pAMPK, which is extremely sensitive to cellular energy levels. The indications of lower metabolic fuel utilization and ATP generation in *Acads−/−* animals, compared to wild type, correspond to previous observations of an energy deficient state [17] in this animal model.

## Additional files

Additional file 1: Table S1. Body weight and energy intake for experimental animals used in microarray. (DOCX 14 kb) Additional file 2: Table S2. Primers used for quantitative real-time PCR assays. (DOC 76 kb) Additional file 3: Table S3. Two factor ANOVA table for liver acylcarnitines. (DOC 67 kb) Additional file 4: Figure S1. Quantification of mtDNA in *Acads−/−* and *Acads+/+* liver. Legend: Relative amount of DNA from two mitochondrial markers, cytochrome B (*mt-Cytb*) and cyclo-oxygenase (*mtCo2*) vs. the nuclear markers hemoglobin beta chain complex (*Hbb*) and glucagon (*Gcg*) in *Acads−/−* and *+/+* mice. Data are presented as mean ± S.E. (*n* = 3 per genotype). (PDF 35 kb) Additional file 5: Table S4. Top 25 up- and down-regulated genes. (XLSX 423 kb) Additional file 6: Table S5. Genotype-by-diet interactions affecting gene expression. (XLSX 23 kb) Additional file 7: Table S6. IPA Pathways. (XLSX 15 kb) Additional file 8: Table S7. Predicted activation/inhibition of upstream regulators all categories. (XLSX 18 kb) Additional file 9: Figure S2. Average-linkage clustering of all categories (transcription factors, enzymes, chemokines, etc.) of candidate upstream regulators identified as significantly activated or inhibited in at least one of the four comparisons (|z|≥2). Clustering results are presented as a heat-map and are color coded according to the magnitude of the z-score. Positive z-scores are depicted in shades of red and negative z-scores in green. (PDF 262 kb)
