# Supplementary material for: Short chain acyl-CoA dehydrogenase deficiency and short-term high-fat diet perturb mitochondrial energy metabolism and transcriptional control of lipid-handling in liver
Source: Nutr Metab (Lond). 2016 Mar 1;13:17. doi: 10.1186/s12986-016-0075-0 (PMC4772307; doi:10.1186/s12986-016-0075-0)
Supplement: Additional file 1: Table S1. — Body weight and energy intake for experimental animals used in microarray. (DOCX 14 kb) [file 12986_2016_75_MOESM1_ESM.docx]

Table S1. *Body weight and energy intake data for animals used in microarray experiment*

|  | *Acads-/-* | *Acads+/+* | HF | LF |
| --- | --- | --- | --- | --- |
| Baseline BW, g | 28.8 ± 0.7^a^ | 29.2 ± 0.4^a^ | - | - |
| HF diet, kcal | 27 ± 1^a^ | 27 ± 1^a^ | - | - |
| LF diet, kcal | 26 ± 1^a^ | 25 ± 2^a^ | - | - |
| *Acads-/-* | - | - | 27 ± 1^a^ | 26 ± 1^a^ |
| *Acads+/+* | - | - | 27 ± 1^a^ | 25 ± 2^a^ |

2 d sums of kcal consumed from either high-fat (HF) or low-fat (LF) diet by *Acads-/-* or *Acads+/+* mice (n=3 per diet and genotype group). BW = body weight. Values with common letters were not significantly different using the criterion *P* < 0.05.
