# Supplementary material for: Short chain acyl-CoA dehydrogenase deficiency and short-term high-fat diet perturb mitochondrial energy metabolism and transcriptional control of lipid-handling in liver
Source: Nutr Metab (Lond). 2016 Mar 1;13:17. doi: 10.1186/s12986-016-0075-0 (PMC4772307; doi:10.1186/s12986-016-0075-0)
Supplement: Additional file 2: Table S2. — Primers used for quantitative real-time PCR assays. (DOC 76 kb) [file 12986_2016_75_MOESM2_ESM.doc]

**Table S2. Gene expression primers**

| **Gene symbol** | **Accession #** | **Forward primer - sequence** | **Position** | **Reverse primer -sequence** | **Position** | **AE** |
| --- | --- | --- | --- | --- | --- | --- |
| Aacs | NM_030210 | 5'-TGGACAGGATAGGGATCACCAT-3' | 1109 | 5'-TGAGTTTCCACTGGCTTCATGT-3' | 1196 | 1.90 |
| Abcc3 | NM_029600 | 5'-GGCGCTGGTGACAAAGACTAA-3' | 2778 | 5'-CTTCACATTGCCTGTCTCTGCTA-3' | 2850 | 1.85 |
| Acaa1a | NM_130864 | 5'-AAGCAGGGCTGACTGTGAATG-3' | 992 | 5'-TCACCTTCTCTGCAGGAATTCC-3' | 1099 | 1.72 |
| Acot1 | NM_012006 | 5'-CGATGACCTCCCCAAGAACA-3' | 579 | 5'-CCCAAGCAGCCCAATTCC-3' | 690 | 1.85 |
| Apol9b | NM_001168660 | 5'-GGCAGGCGTTTGTATCCAA-3' | 170 | 5'-TGTCAGTTCCCTCAGAGCTTTG-3' | 246 | 1.90 |
| Decr2 | NM_011933 | 5'-AAGACCGTGGTTGACATTGACA-3' | 391 | 5'-CATGGTCCCGGAAGAACTTC-3' | 469 | 1.82 |
| Dhrs13 | NM_183286 | 5'-GGCACTGGTGTTACCTGCTATG-3' | 649 | 5'-GCAGCCATCCAGGAAGATGA-3' | 730 | 1.93 |
| Ehhadh | NM_023737 | 5'-TTCAACAGATGAAGCACTCAAGCT-3' | 468 | 5'-GGTTTACCTATAACCGTCTGAGCAA-3' | 569 | 1.81 |
| Elovl6 | NM_130450 | 5'-TCAGCAAAGCACCCGAACTA-3' | 353 | 5'-GGTGGTACCAGTGCAGGAAGA-3' | 433 | 1.67 |
| Fasn | NM_007988 | 5'-GGCACTGACTGTCTGTTTTCCA-3' | 8129 | 5'-TGTAAAAATGACACAGTCCAGACACTT-3' | 8228 | 1.86 |
| Gpam | NM_008149 | 5'-CACAGGCAGGGAATCCATCT-3' | 1486 | 5'-GTCGAAATCGCGAGCTAGGA-3' | 1566 | 1.85 |
| Gpi1 | NM_008155 | 5'-GGGAGAAGCTGGTCTGGAACT-3' | 1798 | 5'-AAGCCCTTCCATCAGCTTCA-3' | 1867 | 1.88 |
| Gpr146 | NM_030258 | 5'-GGTCCATGTCCTCACAAGCA-3' | 1285 | 5'-CGCTTCATTTAGAAAGCCCAAA-3' | 1350 | 1.98 |
| Gstm2 | NM_008183 | 5'-GCACAACCTGTGTGGAGAGACA-3' | 249 | 5'-CATAGCCTGGTTCTCCAAAATGT-3' | 315 | 1.93 |
| Macrod2 | NM_001013802 | 5'-AGCATGTACCCCAGCAACAAG-3' | 232 | 5'-TTCTTTGCGTCTCTCCTCTAAGGT-3' | 321 | 1.79 |
| Mod1 | NM_007622 | 5'-GTGACCAAGGGACGTGCAA-3' | 1267 | 5'-GGGAGAGTGACTGGATCAAAAGG-3' | 1325 | 1.91 |
| Nars | NM_001142950 | 5'-TGAATCTGTGGACGTGTTGATG-3' | 1410 | 5'-CTTCCCTCTTATAGCCTTCGAGAA-3' | 1510 | 1.70 |
| Pdhb | NM_024221 | 5'-ATTCCGAGGATGCAAAAGGA-3' | 536 | 5'-CATTTTCTAGCATTACCACTGGGTTA-3' | 607 | 1.68 |
| Pdk1 | NM_172665 | 5'-AGCACCACGCGGACAAA-3' | 863 | 5'-TCACTCATCTTCACAGTCAGATCCT-3' | 947 | 1.97 |
| Pdk4 | NM_013743 | 5'-CCCTTTGGCTGGTTTTGGT-3' | 975 | 5'-TGATAGCGTCTGTCCCATAACCT-3' | 1084 | 1.92 |
| Pklr | NM_013631 | 5'-TGGAGAGACCGCCAAAGG-3' | 1215 | 5'-ACAACTGGCGGTGGTACACA-3' | 1312 | 1.85 |
| Rps6kl1 | NM_146244 | 5'-GGACCTCAACCCACAGAACCT-3' | 1218 | 5'-CCAACCTCTGGAGCACTGTACA-3' | 1352 | 2.01 |
| Scd1 | NM_009127 | 5'-AAGGGCGGAAAACTGGACAT-3' | 574 | 5'-GCTTGTAGTACCTCCTCTGGAACAT-3' | 646 | 1.98 |
| Slc17a4 | NM_177016 | 5'-TTGCCAGCCTCTGTGATTCA-3' | 1205 | 5'-CCAGCTAGGTAAGAAAAGACTTGCA-3' | 1310 | 1.98 |
| Slc27a5 | NM_009512 | 5'-CAAACAGGGTTTTTGCATTCCT-3' | 1434 | 5'-AGGAAGGGTTGGTTCTTTCGAA-3' | 1514 | 1.88 |
| Sucnr1 | NM_032400 | 5'-CATGGGAGACCATTACAGAGAGATG-3' | 876 | 5'-CCTGAAGGATGTAAGGGACTTGA-3' | 948 | 1.90 |
| Uox | NM_009474 | 5'-TGAGCCAGCTTCCTGAGATAGAA-3' | 737 | 5'-TCAGTCCCATTTTGGACATGTC-3' | 823 | 1.88 |

mtDNA primers

| **Gene symbol** | **Forward primer - sequence** | **Position** | **Reverse primer -sequence** | | **Position** |
| --- | --- | --- | --- | --- | --- |
| mt-Co2 | GCCGACTAAATCAAGCAACA | +530 coding | | CAATGGGCATAAAGCTATGG | +628 coding |
| mt-Cybt | CATTTATTATCGCGGCCCTA | +557 coding | | TGTTGGGTTGTTTGATCCTG | +627 coding |
| Gcg | CAGGGCCATCTCAGAACC | Intron 1 position +1479 chromosome 2 | | GCTATTGGAAAGCCTCTTGC | Intron 1 position +1575 chromosome 2 |
| Hbb | GAAGCGATTCTAGGGAGCAG | Intron 2 position +75 chromosome 7 | | GGAGCAGCGATTCTGAGTAGA | Intron 2 position +164 chromosome 2 |
