# Supplementary material for: Short chain acyl-CoA dehydrogenase deficiency and short-term high-fat diet perturb mitochondrial energy metabolism and transcriptional control of lipid-handling in liver
Source: Nutr Metab (Lond). 2016 Mar 1;13:17. doi: 10.1186/s12986-016-0075-0 (PMC4772307; doi:10.1186/s12986-016-0075-0)
Supplement: Additional file 3: Table S3. — Two factor ANOVA table for liver acylcarnitines. (DOC 67 kb) [file 12986_2016_75_MOESM3_ESM.doc]

**Table S3: Two-factor analysis of variance results for liver acylcarnitines.**

|  | | **Diet** | | **Genotype** | | **Diet x Genotype** | |
| --- | --- | --- | --- | --- | --- | --- | --- |
| **Symbol** | **Biochemical Name** | ***F*** | ***P-value*** | ***F*** | ***P-value*** | ***F*** | ***P-value*** |
| **C2** | Acetyl-L-carnitine | 0.22 | 0.642 | 0.06 | 0.8028 | 1.99 | 0.1721 |
| **C3** | Propionyl-L-carnitine | 24.99 | <0.0001 | 2.47 | 0.1304 | 0.25 | 0.6223 |
| **C4** | Butyryl-L-carnitine | 16.42 | 0.0005 | 114.01 | <0.0001 | 26.59 | <0.0001 |
| **C5** | Valeryl-L-carnitine | 0.23 | 0.6329 | 61.66 | <0.0001 | 0.02 | 0.9036 |
| **C5-DC** | Glutary-L-carnitine | 15.61 | 0.0007 | 0.01 | 0.9188 | 0.27 | 0.6078 |
| **C6** | Hexanoyl-L-carnitine | 3.53 | 0.0736 | 45.61 | <0.0001 | 5.62 | 0.0269 |
| **C8** | Octanoyl-L-carnitine | 1.32 | 0.2631 | 34.92 | <0.0001 | 1.30 | 0.2671 |
| **C8-1** | Octenoyl-L-carnitine | 5.32 | 0.0309 | 29.63 | <0.0001 | 2.95 | 0.1001 |
| **C10** | Decanoyl-L-carnitine | 11.18 | 0.0029 | 29.71 | <0.0001 | 2.53 | 0.1260 |
| **C10-1** | Decenoyl-L-carnitine | 2.46 | 0.1309 | 18.58 | 0.0003 | 0.28 | 0.6020 |
| **C12** | Dodecanoyl-L-carnitine | 41.95 | <0.0001 | 37.36 | <0.0001 | 9.74 | 0.0050 |
| **C12-1** | Dodecanoyl-L-carnitine | 10.99 | 0.0031 | 23.39 | <0.0001 | 3.76 | 0.0653 |
| **C12-OH** | Hydroxy-dodecanoyl carnitine | 48.00 | <0.0001 | 28.14 | <0.0001 | 14.63 | 0.0009 |
| **C14** | Tetradecanoyl-L-carnitine | 4.01 | 0.0576 | 37.42 | <0.0001 | 0.17 | 0.6804 |
| **C14-1** | Tetradecenoyl-L-carnitine | 13.66 | 0.0013 | 20.52 | 0.0002 | 5.44 | 0.0292 |
| **C14-2** | Tetradecadienyl-L-carnitine | 12.49 | 0.0019 | 23.17 | <0.0001 | 4.22 | 0.0520 |
| **C14-OH** | Hydroxy-tetradecanoyl carnitine | 19.61 | 0.0002 | 15.21 | 0.0008 | 2.17 | 0.1550 |
| **C16** | Hexadecanoyl-L-carnitine | 8.00 | 0.0098 | 40.61 | <0.0001 | 2.03 | 0.1685 |
| **C16-1** | Hexadecanoyl-L-carnitine | 13.58 | 0.0013 | 22.04 | 0.0001 | 4.62 | 0.0429 |
| **C16-OH** | Hydroxyhexadecanoyl-L-carnitine | 8.01 | 0.0097 | 7.16 | 0.0138 | 0.05 | 0.8283 |
| **C18** | Octadecanoyl-L-carnitine | 12.93 | 0.0016 | 20.82 | 0.0002 | 0.22 | 0.6401 |
| **C18-1** | Octadecenoyl-L-carnitine | 12.76 | 0.0017 | 32.99 | <0.0001 | 1.56 | 0.2244 |
| **C18-2** | Octadecadienyl-L-carnitine | 0.36 | 0.5563 | 31.40 | <0.0001 | 1.51 | 0.2321 |
| **C18-2OH** | Hydroxyoctadecanoyl-L-carnitine | 3.16 | 0.0892 | 13.67 | 0.0013 | 0.54 | 0.4692 |
| **C20-1** | Eicosatetranoyl-L-carnitine | 31.33 | <0.0001 | 8.14 | 0.0093 | 3.51 | 0.0743 |
| **C20-2** | Eicosatetranoyl-L-carnitine | 19.53 | 0.0002 | 22.20 | 0.0001 | 3.40 | 0.0788 |
| **C20-3** | Eicosatetranoyl-L-carnitine | 5.49 | 0.0286 | 20.23 | 0.0002 | 1.01 | 0.3248 |
| **C20-4** | Eicosatetranoyl-L-carnitine | 0.02 | 0.9002 | 12.11 | 0.0021 | 0.01 | 0.9384 |

Main effects of diet (high-fat, low-fat), genotype (*Acads-/-*, *Acads+/+*), and their interaction on liver acylcarnitine levels. *F*, analysis of variance (ANOVA) *F*-test statistic. *P*, probability of obtaining *F*-test statistic.
