# Supplementary figures and images for: Short chain acyl-CoA dehydrogenase deficiency and short-term high-fat diet perturb mitochondrial energy metabolism and transcriptional control of lipid-handling in liver
Source: Nutr Metab (Lond). 2016 Mar 1;13:17. doi: 10.1186/s12986-016-0075-0 (PMC4772307; doi:10.1186/s12986-016-0075-0)

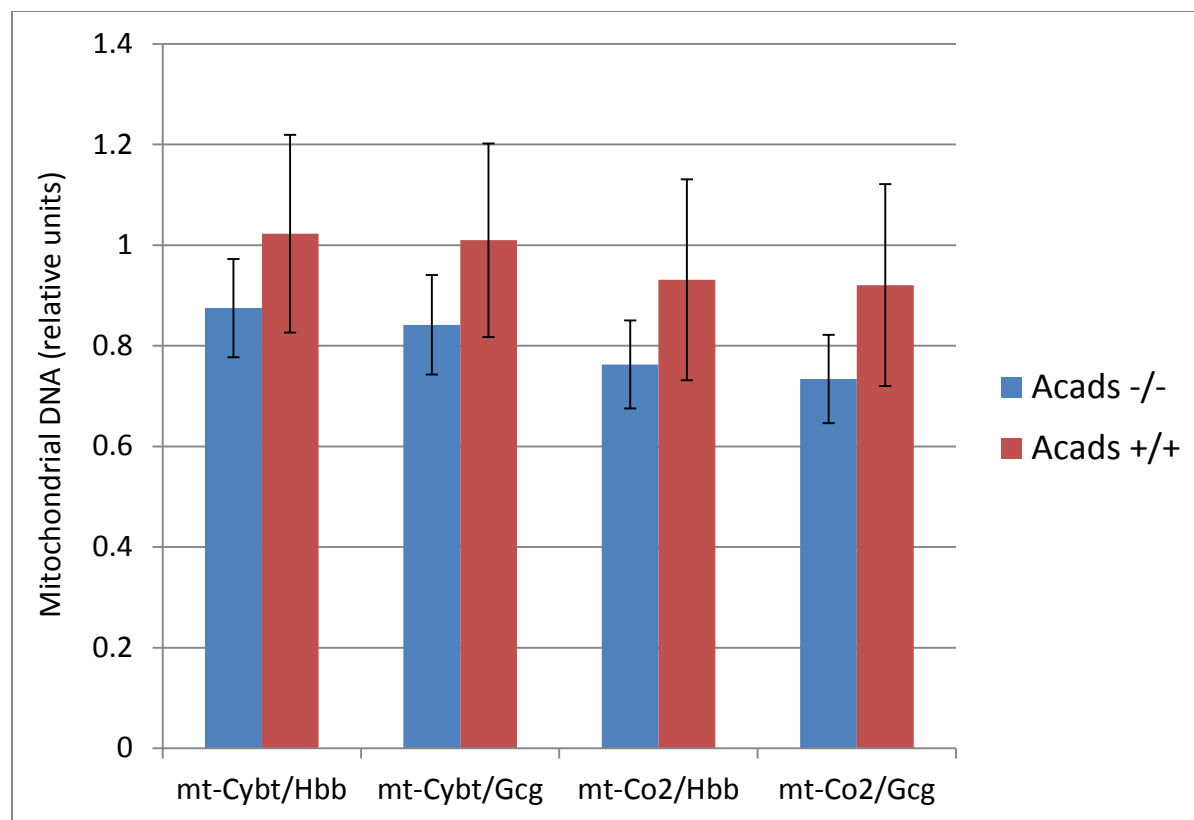

Supplementary Figure

Supplement: Additional file 4: Figure S1. — Quantification of mtDNA in Acads−/− and Acads+/+ liver. Legend: Relative amount of DNA from two mitochondrial markers, cytochrome B (mt-Cytb) and cyclo-oxygenase (mtCo2) vs. the nuclear markers hemoglobin beta chain complex (Hbb) and glucagon (Gcg) in Acads−/− and +/+ mice. Data are presented as mean ± S.E. (n = 3 per genotype). (PDF 35 kb) [file 12986_2016_75_MOESM4_ESM.pdf]
