# Supplementary material for: Short chain acyl-CoA dehydrogenase deficiency and short-term high-fat diet perturb mitochondrial energy metabolism and transcriptional control of lipid-handling in liver
Source: Nutr Metab (Lond). 2016 Mar 1;13:17. doi: 10.1186/s12986-016-0075-0 (PMC4772307; doi:10.1186/s12986-016-0075-0)

Figure S2: Average-linkage clustering of *all categories* of candidate upstream regulators identified as significantly activated or inhibited in at least one of the four comparisons (|z|≥2). Clustering results are presented as a heatmap and color coded according to the magnitude of the z-score. Positive z-scores are colored in shades of red and negative z-scores in green.

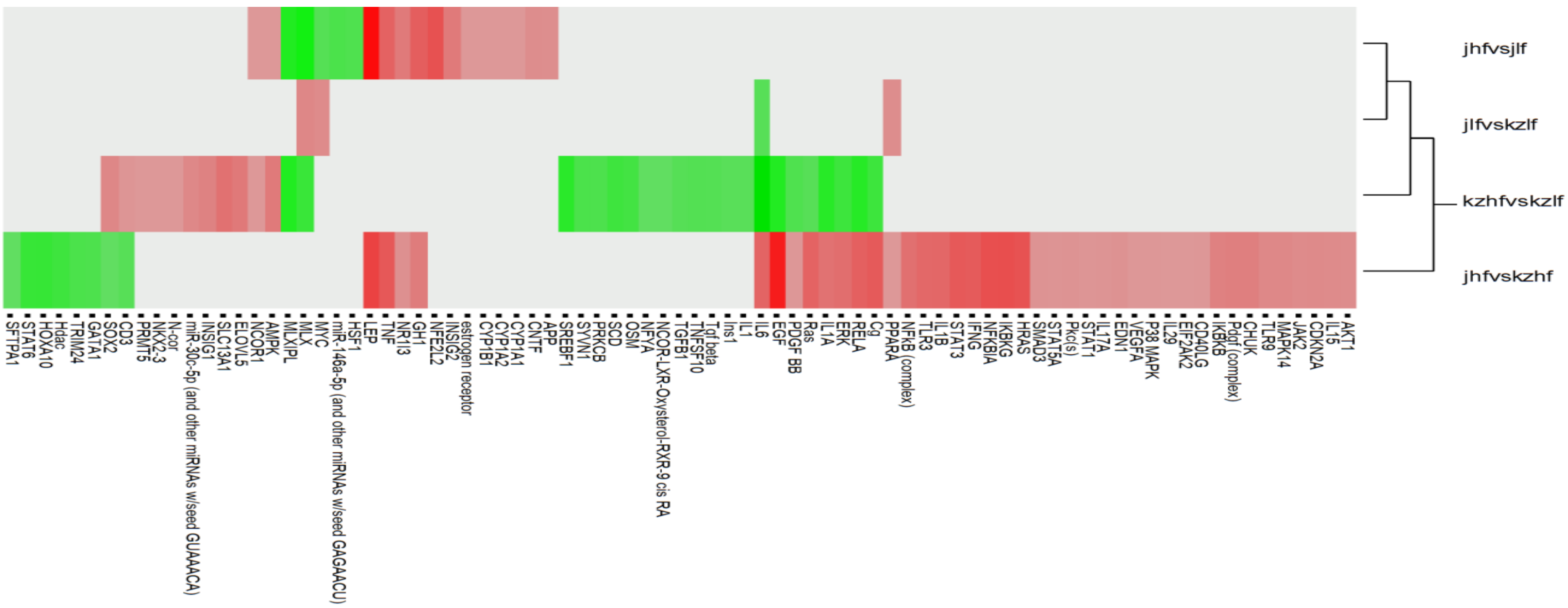

Supplement: Additional file 9: Figure S2. — Average-linkage clustering of all categories (transcription factors, enzymes, chemokines, etc.) of candidate upstream regulators identified as significantly activated or inhibited in at least one of the four comparisons (|z|≥2). Clustering results are presented as a heat-map and are color coded according to the magnitude of the z-score. Positive z-scores are depicted in shades of red and negative z-scores in green. (PDF 262 kb) [file 12986_2016_75_MOESM9_ESM.pdf]
